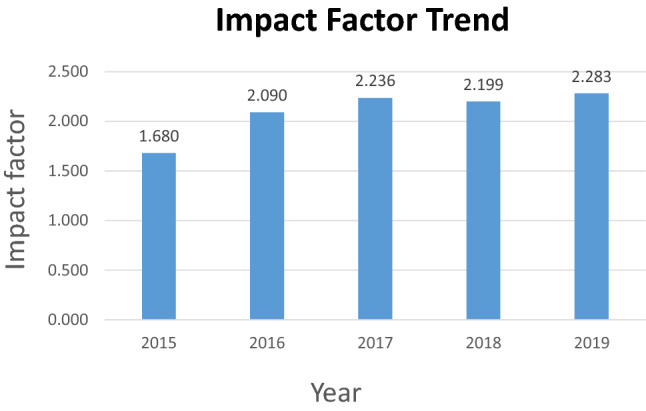# Message from the Editor-in-Chief

**DOI:** 10.1007/s00404-020-05736-7

**Published:** 2020-08-16

**Authors:** O. Ortmann

**Affiliations:** Regensburg, Germany

Dear Reader,

The Editorial Board of *Archives of Gynecology and Obstetrics* has successfully improved the quality of the journal during the last years. The journal receives currently about 2,000 submissions per year from all over the world. Outstanding editors, associate editors, editorial board members, and reviewers have greatly contributed to the successful development of the journal. In addition, the members of the editorial office did a great job in handling the manuscripts. The quality of the scientific work improved a lot. All this led to an increase of the impact factor which is now 2.283.

*Archives of Gynecology and Obstetrics* have a long and outstanding history. In this year, we celebrate the 150th anniversary. The journal was founded in 1870 as“Archiv für Gynäkologie”. Since 1922, it is the official journal of the German Society of Gynecology and Obstetrics (Deutsche Gesellschaft für Gynäkologie und Geburtshilfe, DGGG). In the review by M. David and A. D. Ebert, “Fair to all interests and impartial towards all schools of thought in honour of the 150th anniversary of the foundation of the Archiv/Archives”, published in this issue, the history of *Archives of Gynecology and Obstetrics* is summarized. After the *American Journal of Obstetrics and Gynecology*, our journal is the second oldest journal in this field. You are invited to submit manuscripts for publication in the different sections. We appreciate your suggestions leading to further improvement of the journal's quality.